# Preparation of nanoporous BiVO_4_/TiO_2_/Ti film through electrodeposition for photoelectrochemical water splitting

**DOI:** 10.1098/rsos.180728

**Published:** 2018-09-19

**Authors:** Dong Hongxing, Liu Qiuping, He Yuehui

**Affiliations:** 1 College of Electromechanical Engineering, Hangzhou Polytechnic, Hangzhou, Zhejiang, People's Republic of China; 2 College of Chemical Engineering, Zhejiang University of Technology, Hangzhou, Zhejiang, People's Republic of China; 3 State Key Laboratory of Powder Metallurgy, Central South University, Changsha 410083, People's Republic of China

**Keywords:** BiVO_4_/TiO_2_/Ti photoelectrodes, electrodeposition, photocurrent density, water splitting

## Abstract

A nanoporous BiVO_4_/TiO_2_/Ti film was successfully fabricated by electrodepositing a nanoporous BiOI film on nanoporous TiO_2_ arrays followed by annealing at 450°C for 2 h. The electrodeposition of BiOI film was carried out at different times (10, 30, 100, 500 and 1000 s) in Bi(NO_3_)_3_ and KI solution. The morphological, crystallographic and photoelectrochemical properties of the prepared BiVO_4_/TiO_2_/Ti heterojunction film were examined by using different characterization techniques. UV–vis spectrum absorption studies confirmed an increase in absorption intensities with increasing electrodeposition time, and the band gap of BiVO_4_/TiO_2_/Ti film is lower than that of TiO_2_/Ti. The photocatalytic efficiency of BiVO_4_/TiO_2_/Ti heterojunction film was higher compared to that of the TiO_2_/Ti film owing to the longer transient decay time for BiVO_4_/TiO_2_/Ti film (3.2 s) than that of TiO_2_/Ti film (0.95 s) in our experiment. The BiVO_4_/TiO_2_/Ti heterojunction film prepared by electrodeposition for 1000 s followed by annealing showed a high photocurrent density of 0.3363 mA cm^−2^ at 0.6 V versus saturated calomel electrode. Furthermore, the lowest charge transfer resistance from electrochemical impedance spectroscopy was recorded for the BiVO_4_/TiO_2_/Ti film (1000 s) under irradiation.

## 1. Introduction

In the past few decades, photoelectrochemical (PEC) catalytic water splitting by using nanostructured semiconductors has been an effective way of producing hydrogen and oxygen [1,2]. Many types of photoelectrode materials have been reported, including BiVO_4_, CuO and Fe_3_O_4_ [3–6]. Among these materials, BiVO_4_ has attracted significant attention because of its high photocatalytic activity in oxygen evolution under an external bias. However, the conduction band (CB) level is low and it is unsuitable for O_2_ formation. The high-energy electrons excited by light usually relax in the bottom of the CB quickly owing to its weak charge-carrier separation [7,8].

Recently, there are two types of modification methods to further improve the photocatalytic ability of the semiconductor photocatalyst of BiVO_4_. One is doping modification, such as doped metal [9], non-metal [10] and semiconductor [11]. The second method is morphology modification by changing the crystal structure, morphology and specific surface area of BiVO_4_ [12,13]. The heterogeneous catalytic materials combining with BiVO_4_ are the most promising structures owing to their uniqueness and excellent coupling ability. There are many types of heterogeneous materials including WO_3_/BiVO_4_, g-C_3_N_4_/BiVO_4_ and TiO_2_/BiVO_4_ [14,15].

Among these heterogeneous materials, BiVO_4_/TiO_2_ remains one of the best photocatalytic materials because of the low cost of TiO_2_ and the faster electron transfer properties. Moreover, the TiO_2_ membrane has great potential in developing highly efficient water treatment and reuse systems, such as the decomposition of organic pollutants [16]. Concerning BiVO_4_/TiO_2_, it has been extensively reported for photo-oxidative degradation of pollutants, such as rhodamine [17,18]. In addition, most of the BiVO_4_/TiO_2_ electrodes are prepared through sol–gel, metal organic decomposition, chemical thermal deposition and spinning [17,19–21]. An efficient charge transfer between BiVO_4_ and TiO_2_ has already been reported [22]. Nanoporous TiO_2_/Ti arrays have been successfully prepared by anodic oxidation and the electrocatalytic properties have also been extensively analysed [23]. However, few studies that put porous BiVO_4_ film onto nanoporous TiO_2_/Ti arrays through electrodeposition of BiOI film followed by a sintering method have been conducted.

Therefore, the present work adds useful information on heterogeneous materials. BiVO_4_/TiO_2_/Ti photoanodes were fabricated by electrodepositing BiOI film on nanoporous TiO_2_ arrays followed by annealing at 450°C. PEC characterization of BiVO_4_/TiO_2_/Ti heterojunction material with different BiVO_4_ thicknesses was first performed in 0.2 M Na_2_SO_4_ electrolyte under an Xe lamp irradiation using CHI660E, which will provide new insights on further works in the field of BiVO_4_-based heterogeneous materials.

## 2. Experimental procedure

### 2.1. Preparation of nanoporous TiO_2_/Ti film

Commercially pure titanium plates (0.5 mm thick, purity greater than 99.5%) were first degreased in acetone, mechanically polished, and finally, chemically polished at 25°C in a solution consisting of H_2_O : HNO_3_ : HF = 6 : 3 : 1 (vol%) for 30 s. The pretreated titanium plates were anodically oxidized at 25°C in 1 wt% hydrofluoric acid at 20 V for 15 min to produce nanoporous TiO_2_/Ti arrays. Then the nanoporous TiO_2_ arrays were washed three times with de-ionized water and dried for the next step usage. The prepared nanoporous TiO_2_ arrays were sintered in air at 450°C for 2 h to prepare the nanoporous TiO_2_/Ti film.

### 2.2. Preparation of BiVO_4_/TiO_2_/Ti heterojunction photoanodes

The BiOI film was successfully prepared by electrodeposition [24,25]. The 0.04 M Bi(NO_3_)_3_ solution was prepared by dissolving Bi(NO_3_)_3_·5H_2_O in 50 ml 0.4 M KI solution after its pH was adjusted to 2.0 by adding HNO_3_. Then the solution was mixed with 20 ml absolute ethanol (100%) containing 0.23 M *p-*benzoquinone and was vigorously stirred for 15 min. A typical three-electrode cell was used for electrodeposition. Nanoporous TiO_2_/Ti film, saturated calomel electrode (SCE) and Pt silk were used as the working electrode, reference electrode and counter electrode, respectively. CHI660E (Shanghai Chenhua Device Company, China) was used for electrodeposition and subsequent PEC studies. Electrodeposition was performed potentiostatically at −0.15 V versus SCE at room temperature with varying deposition times (10, 30, 100, 500 and 1000 s). Then the 0.2 M vanadyl acetylacetonate (VO (acac)_2_) dissolved in dimethyl sulfoxide was drop-cast onto the working electrode with a volume of 1 ml followed by heating in a muffle furnace at 450°C for 2 h in air. After that, the excess V_2_O_5_ on the BiVO_4_/TiO_2_/Ti photoelectrodes was removed in 1 M NaOH solution for 1–2 h with stirring [24]. The resulting heterojunction BiVO_4_/TiO_2_/Ti photoelectrodes were then rinsed with de-ionized water and dried at room temperature.

### 2.3. Characterization

The surface morphologies of heterojunction BiVO_4_/TiO_2_/Ti photoanodes were observed by Hitachi S-4700 field emission scanning electron microscopy (FESEM) after spraying the conducting layer with platinum. The bulk composition was investigated by energy-dispersive X-ray spectroscopy. The phases present in the coatings were characterized by a small angle diffractometric study carried out on a Riga KuD/max 2550PC X-ray automatic diffractometer. The optical performance of the as-prepared materials was evaluated by using a UV–vis Lambda 750S in a wavelength ranging from 300 to 800 nm.

The PEC performance was evaluated in a three-electrode electrochemical cell with a quartz window to allow illumination. The working electrodes were the sintered nanoporous TiO_2_/Ti film and BiVO_4_/TiO_2_/Ti heterojunction film. SCE and Pt silk were used as the reference electrode and counter electrode, respectively. All the working electrodes were characterized in 0.2 M Na_2_SO_4_ by CHI660E. Linear sweep voltammetry (LSV) was measured at a scanning rate of 0.01 V s^−1^. Electrochemical impedance spectroscopy (EIS) was carried out under an open circuit voltage with frequencies ranging from 10^5^ to 10^−2^ Hz with an AC voltage amplitude of 5 mV. The potentials in the I–V curves and in the PEC performance experiments were also controlled by CHI660E. A 150 W Xe lamp (Beijing Trust Tech Co. Ltd) was used to provide the visible light. EIS was used to explore the conductivity of the as-compared electrodes in dark and illumination environments in 0.2 M Na_2_SO_4_ solution.

## 3. Results and discussion

**Figure 1.**
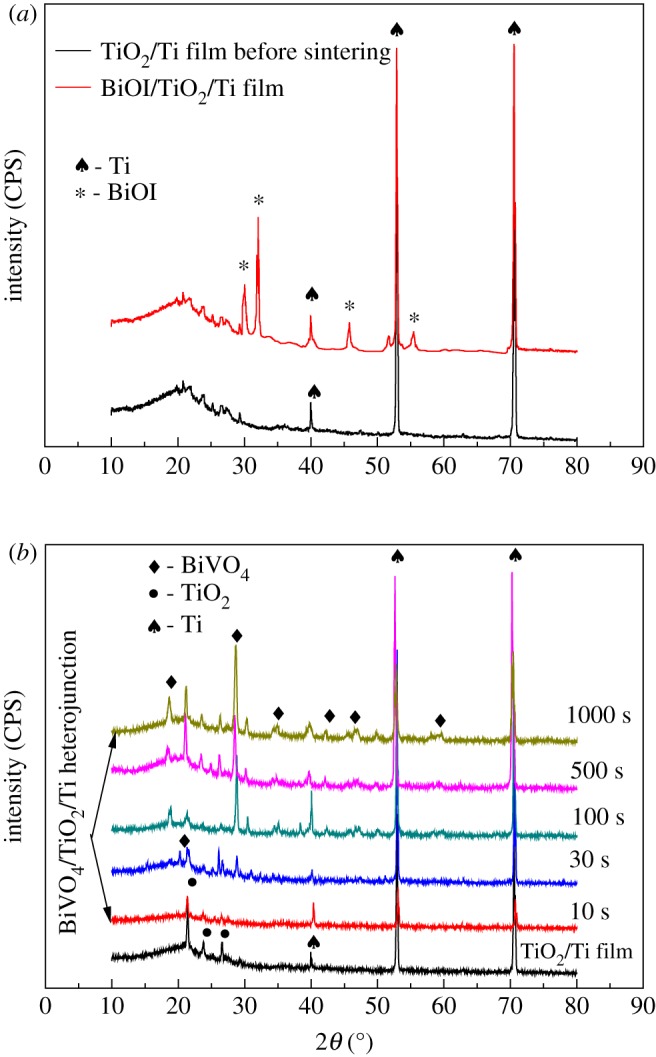
XRD patterns: (*a*) TiO_2_/Ti before sintering and BiOI/TiO_2_/Ti with 1000 s electrodeposition time; (*b*) sintered nanoporous TiO_2_/Ti film and BiVO_4_/TiO_2_/Ti heterojunction films prepared with different electrodeposition times.

The crystal structures of sintered nanoporous TiO_2_/Ti film and BiVO_4_/TiO_2_/Ti heterojunction films prepared with different electrodeposition times were characterized by X-ray diffraction (XRD) and are shown in figure 1. In figure 1*a*, the diffraction peaks at 2*θ* of 29.6°, 31.6°, 45.3° and 51.3° can be indexed to BiOI (JCPDS no. 10–0445). The as-anodized nanoporous TiO_2_/Ti films are of amorphous state due to the broad peak and only the peaks of the titanium substrate are present in the diffractogram [26]. After sintering at 450°C for 2 h, peaks at approximately 25° appear corresponding to anatase phase [26]. After modifying by BiVO_4_ layer, new peaks appear at 2*θ* of 18.6°, 18.9°, 28.7°, 34.4°, 35.2°, 42.3°, 46.4° and 58.3°, which correspond to (101), (011), (-121), (200), (002), (051), (002), (202) and (321) of BiVO_4_. The observed diffraction peaks are in conformity with monoclinic scheelite structure (JCPDS 14-0688). The XRD patterns confirm the full conversion of BiOI to BiVO_4_ after annealing at 450°C. From the XRD pattern, it was clarified that BiVO_4_ could be successfully modified on nanoporous TiO_2_/Ti films.

**Figure 2.**
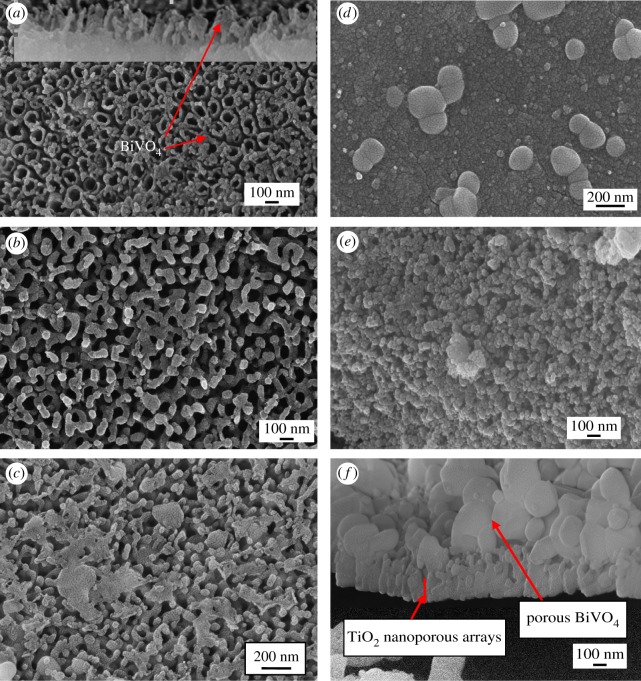
SEM images of photoelectrodes prepared with different electrodeposition times: (*a*) surface image of electrode with 10 s electrodeposition time; inset picture is the cross-section morphology prepared by mechanical fracturing; (*b*) surface image of electrode with 30 s electrodeposition time; (*c*) surface image of electrode with 100 s electrodeposition time; (*d*) surface image of electrode with 500 s electrodeposition time; (*e*) surface image of electrode with 1000 s electrodeposition time; (*f*) cross-section of sample (*e*) with mechanical fracturing.

The morphology and nanostructures of BiVO_4_/TiO_2_/Ti heterojunction photoanodes were characterized using FESEM. Here, the morphology of TiO_2_/Ti film was not shown, because its morphology could be easily seen from the morphology of BiVO_4_/TiO_2_/Ti heterojunction photoanodes obtained with 10 s electrodeposition time. From the top view of the anode with 10 s electrodeposition time (figure 2*a*), it is found that a small amount of BiVO_4_ existed in the interstice between the TiO_2_ nanotubes and on the top of TiO_2_ nanoporous wall. This is confirmed by the cross-section image in the inset picture of figure 2*a*. The TiO_2_ nanotube arrays are formed with regular and orderly structure. The ratio of pore length to diameter was approximately 2 : 1. When the electrodeposition time increased to 30 s, BiVO_4_ covered the interstices between the TiO_2_ walls and left the TiO_2_ pores open. Some of the TiO_2_ nanopores were covered by BiVO_4_ when the electrodeposition time increased to 100 s (figure 2*c*). BiVO_4_ covered the TiO_2_ nanopores and particles of approximately 200 nm grew on the surface when the electrodeposition time increased to 500 s (figure 2*d*). When the electrodeposition time increased to 1000 s, the morphology of the obtained photoanode was nanoporous and the thickness of BiVO_4_ was approximately 1 µm. Compared with figure 2*d*,*f*, the particle size of BiVO_4_ is almost 100 nm. This phenomenon is different from the conventional electrodeposition procedure in which the particle size usually increases with electrodeposition time [27].

**Figure 3.**
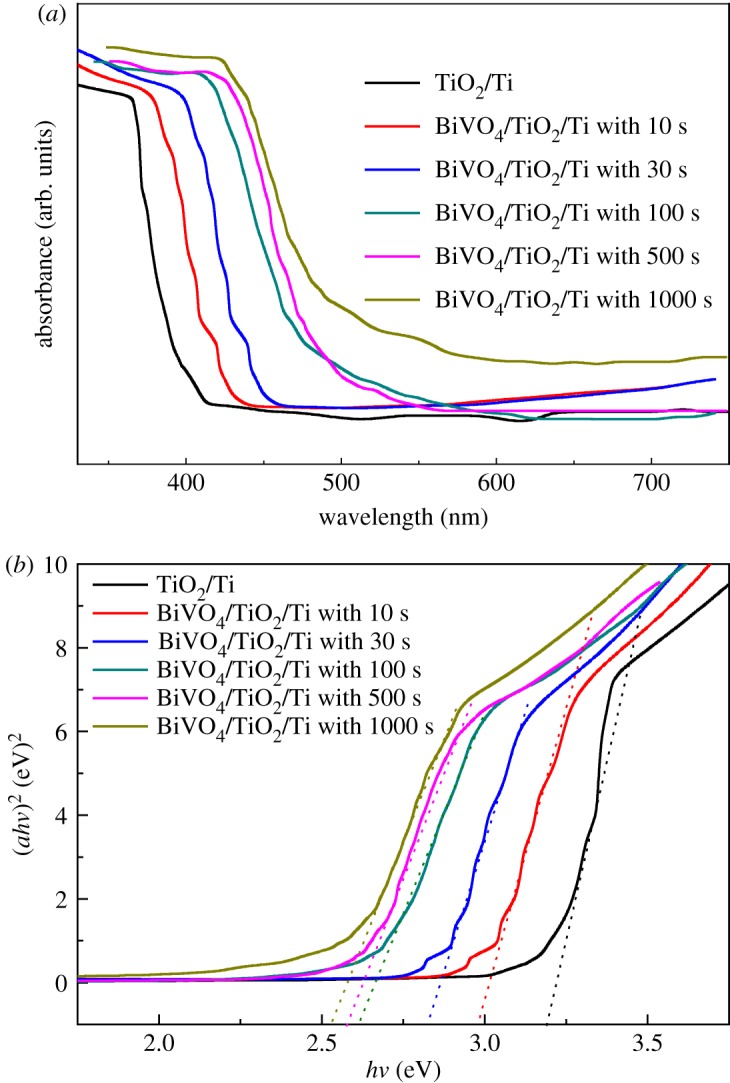
(*a*) UV–vis absorption spectra and (*b*) Tauc plot of nanoporous TiO_2_/Ti film and BiVO_4_/TiO_2_/Ti heterojunction films prepared with different electrodeposition times.

The UV–vis spectra of TiO_2_/Ti film and BiVO_4_/TiO_2_/Ti heterojunction films with different amounts (denoted by the electrodeposition time) of BiVO_4_ are shown in figure 3*a*. The absorption edge of the annealed TiO_2_/Ti film is approximately 375 nm. After being coupled with BiVO_4_, the absorption in the visible light region between 400 and 500 nm of BiVO_4_/TiO_2_/Ti heterojunction film increases with the increase in the amount of BiVO_4_, and the absorption edge shifts to 450 nm for the BiVO_4_/TiO_2_/Ti film prepared with 1000 s electrodeposition time. The band gap of a semiconductor can be calculated by employing the following equation [28]:

3.1 $${\left(ahv\right)}^{2}=hv-E_{g},$$

where *a* is the absorption coefficient, *v* is the light frequency and *E*_g_ is the band gap of a semiconductor. From the curve of (*ahv*)^2^ versus *hv* shown in figure 3*b*, the energy of the band gap of TiO_2_/Ti film is 3.20 eV, which is the same as the result reported before [29]. The band gap energies of the BiVO_4_/TiO_2_/Ti film with 10, 30, 100, 500 and 1000 s electrodeposition times are calculated as 2.98, 2.83, 2.62, 2.58 and 2.53 eV, respectively. All BiVO_4_/TiO_2_/Ti films exhibited visible-light-driven absorption characteristics. Moreover, the nanoporous BiVO_4_ film in the BiVO_4_/TiO_2_/Ti film has slightly shifted on the absorption edge to the larger wavelength within the visible-light range owing to the effect of additional BiVO_4_.

**Figure 4.**
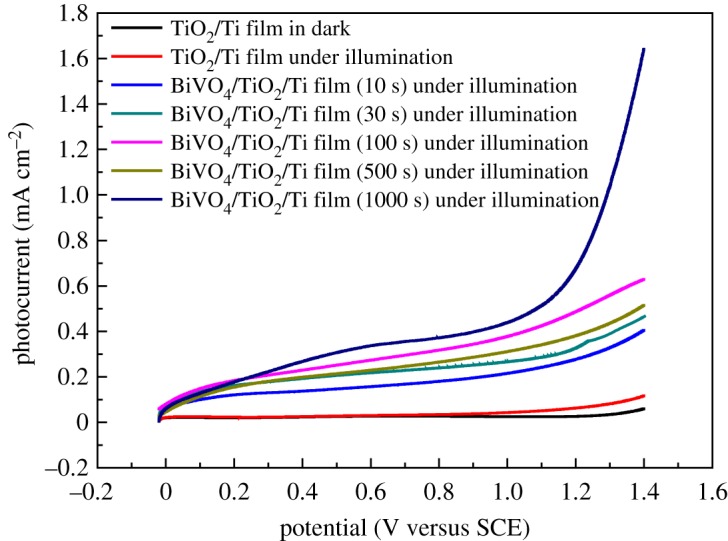
LSV plots of nanoporous TiO_2_/Ti film and nanoporous BiVO_4_/TiO_2_/Ti films prepared with different electrodeposition times in 0.2 M Na_2_SO_4_ solution under 150 W Xe lamp illumination.

Figure 4 shows the current–potential plots of nanoporous TiO_2_/Ti film and nanoporous BiVO_4_/TiO_2_/Ti heterojunction photoanodes under a 150 W Xe lamp illumination. The photocurrent densities of nanoporous TiO_2_/Ti film in the dark and under illumination were 0.02634 mA cm^−2^ and 0.0308 mA cm^−2^ at 0.6 V (versus SCE), respectively. The BiVO_4_/TiO_2_/Ti prepared with 10 s electrodeposition time under illumination exhibited a higher photocurrent density of 0.1583 mA cm^−2^ at 0.6 V (versus SCE) and showed a 403% higher photoactivity compared with bare TiO_2_/Ti film. Moreover, the photocurrent density of BiVO_4_/TiO_2_/Ti heterojunction photoanode increased with the increase in electrodeposition time in our experiment. When the electrodeposition time increased to 1000 s, the photocurrent density increased to 0.3363 mA cm^−2^ at 0.6 V (versus SCE). These results clearly indicate that the modification of nanoporous TiO_2_/Ti film with BiVO_4_ effectively reduces the recombination of electrons and holes generated in the nanoporous BiVO_4_/TiO_2_/Ti film due to the formation of the heterojunction and excellent electron transport between TiO_2_ film and the titanium substrate [30]. When BiVO_4_ layers were coated on the TiO_2_ surface, the light absorption range and intensity of BiVO_4_/TiO_2_/Ti films were improved and the electrons of BiVO_4_ film could easily transfer to the nanoporous TiO_2_, resulting in a high photocurrent density.

**Figure 5.**
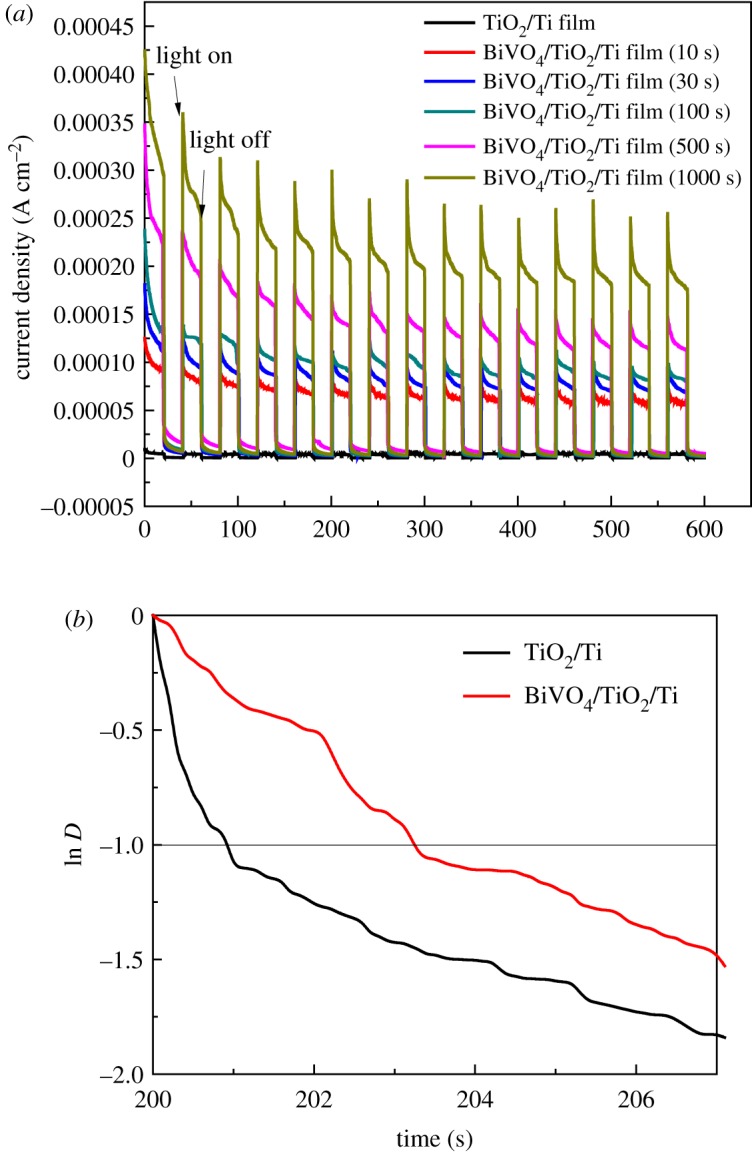
(*a*) Transient photocurrent responses of TiO_2_/Ti film and BiVO_4_/TiO_2_/Ti heterojunction film prepared with different electrodeposition times under 150 W Xe lamp illumination in 0.2 M Na_2_SO_4_ solution at 0.6 V versus SCE. (*b*) Transient decay times of TiO_2_/Ti film and BiVO_4_/TiO_2_/Ti heterojunction film.

The photocurrent response of photoanodes in the electrolyte directly correlates with the generation and transfer of the photo-excited charge carriers in the photocatalytic process [31]. The photocurrent responses of TiO_2_/Ti film and BiVO_4_/TiO_2_/Ti heterojunction films prepared with different electrodeposition times were investigated to enhance the charge separation in 0.2 M Na_2_SO_4_ electrolyte at 0.6 V bias versus SCE. Both TiO_2_ and BiVO_4_ absorbed the photons and generated electron-hole pairs under the simulated sunlight illumination [32]. As shown in figure 5, it is clear that the photocurrent abruptly increased and decreased when the light source was switched on and off. The photoanodes of BiVO_4_/TiO_2_/Ti heterojunction films present obviously enhanced the photocurrent response compared with those of bare TiO_2_/Ti film. A photocurrent spike is clearly obtained in sudden illumination due to capacitive charging of the interface, and the spike decays because of recombination of the charge carriers associated with holes getting trapped at the surface [33]. When the simulated sunlight was turned on, the photocurrent density was a little higher than that 10 s later, which indicated the poor recombination abilities of photogenerated electrons with the holes in the TiO_2_ modified with BiVO_4_ electrodes [34]. It is obvious that the BiVO_4_/TiO_2_/Ti heterojunction films with 1000 s electrodeposition time represent the highest photocurrent density compared to that of other photoanodes, which can be ascribed to the high nanoporous surface of BiVO_4_/TiO_2_/Ti heterojunction photoanodes and their excellent charge separation and transport properties. Thus, it can also be confirmed that the separation of electron-hole pairs was derived from the heterojunction [35].

The transient decay time can be analysed by a logarithmic plot of parameter *D*, using the following equation [33,36]:

3.2 $$D=\frac{I_{t}-I_{s}}{I_{m}-I_{s}},$$

where *I_t_* is the current at time *t*, *I_s_* the stabilized current and *I_m_* is the current spike. The transient decay time can be defined as the time at which ln *D* = −1 [37]. Figure 5*b* displays the logarithmic plots of parameter *D* of the BiVO_4_/TiO_2_/Ti heterojunction film prepared with 10 s and TiO_2_/Ti photoelectrodes. The transient decay time for BiVO_4_/TiO_2_/Ti (3.2 s) is longer than that of TiO_2_/Ti (0.95 s), indicating a lower charge carrier recombination rate in BiVO_4_/TiO_2_/Ti heterojunction photoanodes, leading to an enhanced charge separation efficiency and prolonging the hole lifetimes. The BiVO_4_/TiO_2_/Ti electrodes prepared with increasing electrodeposition time maintained an improving PEC performance.

**Figure 6.**
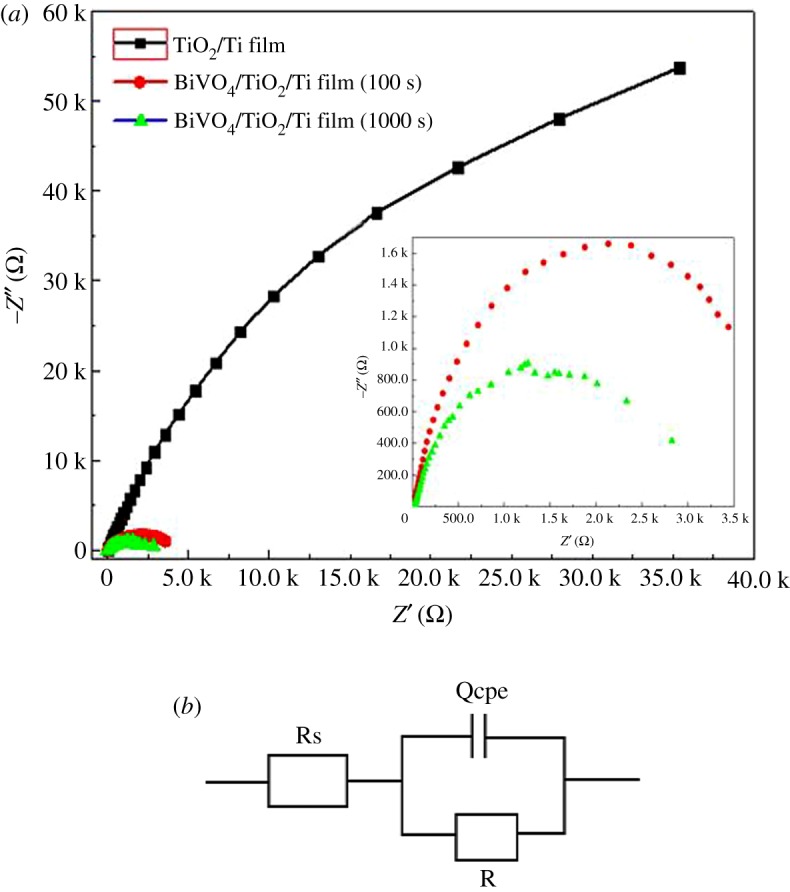
(*a*) EIS spectra of TiO_2_/Ti film and BiVO_4_/TiO_2_/Ti heterojunction film prepared with different electrodeposition times under 150 W Xe lamp illumination in 0.2 M Na_2_SO_4_ solution at 0.6 V versus SCE. (*b*) Equivalent circuit for photoanodes.

To evaluate the kinetics of the charge transfer process of the TiO_2_/Ti and BiVO_4_/TiO_2_/Ti photoelectrodes, EIS tests were carried out at 0.2 V versus SCE under a simulated solar light illumination. Figure 6*a* displays the Nyquist diagrams in the frequency range of 0.01 Hz to 100 kHz. In the plot, symbols indicate the experimental results and the inset picture is the magnified view of the Nyquist diagram of BiVO_4_/TiO_2_/Ti heterogeneous photoanodes. The arc in the Nyquist plot indicates the charge transfer kinetics on the working electrode. Obviously, the BiVO_4_/TiO_2_/Ti photoelectrodes present a lower charge transfer resistance, suggesting that the BiVO_4_/TiO_2_/Ti heterojunction facilitates charge transfer and separation. The simulated EIS results were obtained from the fitting procedures according to the ZSimpWin software, and the equivalent Randles circuit is shown in figure 6*b*. In the equivalent Randles circuit, Rs is the solution resistance, Qcpe is the constant phase element for the electrolyte/electrode interface and R is the charge transfer resistance across the interface of electrode/electrolyte. The arcs in the Nyquist plot are related to the charge transfer at the interface of the photoelectrode/electrolyte. The fitted values of R were 3183, 4373 and 322 000 Ω cm^−2^ for BiVO_4_/TiO_2_/Ti (1000 s), BiVO_4_/TiO_2_/Ti (100 s) and TiO_2_/Ti electrodes, respectively. The efficient charge transfer at the interface between photoelectrode and electrolyte hinders the charge recombination and induces the facile charge transport of electrons through the films. Thus, the bare TiO_2_/Ti film has a very low efficiency of charge transfer and shows the highest R value. The lowest R value for BiVO_4_/TiO_2_/Ti (1000 s) indicates that the charge transfer characteristics of BiVO_4_/TiO_2_/Ti heterojunction are good. Therefore, the modification of TiO_2_/Ti film with nanoporous BiVO_4_ by forming the heterojunction could improve the charge transfer and photocatalytic ability of photoanodes.

**Figure 7.**
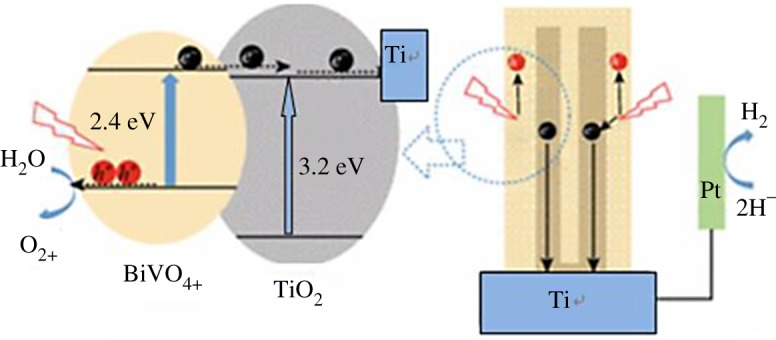
Schematic of energy bands and charge transfers at BiVO_4_/TiO_2_/Ti film.

The photogenerated electrons can move to the CB of TiO_2_ from the CB of BiVO_4_ easily owing to the type II heterojunction. The excited electrons in TiO_2_ were facilely transported by the conductive Ti and directed to the Pt counter electrode via the external circuit (shown in figure 7). Therefore, the photogenerated electrons were scavenged by hydrogen ions on the Pt foil, while the photogenerated holes oxidized the water on the surface of the BiVO_4_/TiO_2_/Ti. The significantly enhanced PEC performance is attributed to the nanoporous structure, which improved the charge transport [38] and collection efficiency as well as the excellent contact between the TiO_2_ and BiVO_4_ with a large interface area facilitating the charge separation. Overall, the BiVO_4_/TiO_2_/Ti heterojunction film offered a remarkable photoconversion efficiency.

## 4. Conclusion

A new type of nanoporous BiVO_4_/TiO_2_/Ti heterojunction photoanode was designed and fabricated by electrodepositing BiOI onto a TiO_2_/Ti nanoporous film followed by sintering at 450°C in vanadium (IV) oxy acetylacetonate solution for 2 h. A significant change was observed in the PEC properties of the BiVO_4_/TiO_2_/Ti heterojunction film by varying the electrodeposition time. The film electrodeposited for 1000 s showed a high photocurrent density of 0.3363 mA cm^−2^ at 0.6 V versus SCE. Furthermore, the lowest charge transfer resistance from electrochemical impedance spectroscopy was recorded for the BiVO_4_/TiO_2_/Ti heterojunction film electrodeposited for 1000 s under irradiation. Our results demonstrate that the nanoporous heterojunction BiVO_4_/TiO_2_/Ti photoanode is an effective design for improving the PEC performance owing to the excellent transport and separation efficiency. It will open a new opportunity for BiVO_4_/TiO_2_/Ti heterojunction photoelectrodes for water splitting by using solar energy.

## Data accessibility

Data available from the Dryad Digital Repository: http://dx.doi.org/10.5061/dryad.d53pg0j [39]
